# Palate to Periosteum: A Multisystem Manifestation of Tuberculosis in an Unusual Distribution

**DOI:** 10.7759/cureus.107735

**Published:** 2026-04-26

**Authors:** Yon Lek Yap, Hans Prakash Sathasivam, Hwee Cheng Chong

**Affiliations:** 1 Rheumatology, Hospital Melaka, Melaka, MYS; 2 Ministry of Health, National Institutes of Health, Selangor, MYS

**Keywords:** disseminated tuberculosis, erosive arthritis, extrapulmonary tuberculosis, oral mass, tuberculous arthritis, ziehl-neelsen stains

## Abstract

Tuberculous (TB) arthritis accounts for approximately 1-3% of TB cases and typically involves large joints, whereas oral TB is extremely rare. The coexistence of an oral ulcerated mass and monoarticular erosive arthritis is unusual and may mimic malignancy, often leading to delayed diagnosis. We report a 50-year-old previously healthy Chinese male patient, who presented with a six-month history of right wrist pain, palatal pain, intermittent fever, anorexia, and significant weight loss, followed by difficulty in chewing and swallowing. Examination revealed cachexia, an ulcerated mass on the hard palate, and synovitis of the right wrist. Investigations showed anemia, elevated inflammatory markers, hypercalcemia, and renal impairment. Wrist radiographs demonstrated erosive changes, while chest radiography showed reticulonodular opacities. Acid-fast bacilli (AFB) were detected in nasogastric lavage and endotracheal aspirate specimens, with subsequent culture confirmation, while histopathological examination of the palatal lesion confirmed TB. Although direct tissue confirmation of the wrist lesion was not obtained, its clinical and radiological features were highly suggestive of musculoskeletal involvement in the context of disseminated disease. Despite initiation of anti-TB therapy, the patient rapidly deteriorated and succumbed to multiorgan failure.

This single case highlights an uncommon presentation of disseminated TB with oral, pulmonary and probable wrist involvement. It reinforces the need for a high index of clinical suspicion and timely tissue biopsy, where feasible, to facilitate early diagnosis and improve patient outcomes.

## Introduction

Tuberculosis (TB), caused by *Mycobacterium tuberculosis*, remains a significant global health concern, particularly in endemic regions. The disease most commonly manifests as pulmonary TB, which is well characterized due to its typical clinical presentation and transmissibility. However, TB is a multisystem infection with the potential to involve virtually any organ.

While pulmonary TB is familiar to most clinicians, extrapulmonary TB (EPTB) is less straightforward and often poses diagnostic challenges due to its variable and non-specific clinical manifestations. Extrapulmonary spread most commonly occurs via hematogenous dissemination from a primary pulmonary focus, leading to seeding of distant organs. Diagnosis of EPTB requires a combination of clinical suspicion, imaging, and microbiological or histopathological confirmation. Bacterial culture for TB remains the gold standard, while molecular testing (PCR) provides important adjunctive support. Obtaining tissue from all affected sites may not always be feasible, necessitating reliance on accessible specimens. Differential diagnoses, particularly malignancy and fungal infections, should always be considered in cases of multisystem disease.

TB arthritis accounts for 1-3% of all TB cases [1]. It typically involves large weight-bearing joints but may occasionally affect smaller, non-weight-bearing joints as well. Oral TB is exceptionally rare compared to oral malignancies, accounting for less than 1% of all EPTB cases. An ulcerated oral mass accompanied by monoarticular erosive arthritis is a rare presentation, often signaling underlying systemic infections (including fungal or granulomatous diseases) or malignancies. 

We report a case from the Rheumatology Unit of Hospital Melaka in which a patient with these manifestations was diagnosed with disseminated TB but unfortunately succumbed to the disease. The patient had pulmonary and oral involvement (confirmed with culture and histopathological examination), with probable monoarticular TB arthritis. 

## Case presentation

A 50-year-old Chinese male with no prior medical history was referred to the Rheumatology unit for evaluation of suspected inflammatory monoarthritis. He had right wrist pain for the past six months, associated with progressive palatal pain, intermittent fever, anorexia, and unintentional weight loss of 10kg over the same duration. He had been self-medicating with over-the-counter analgesics with partial relief. This was followed by two months of difficulty in chewing and swallowing. He denied respiratory or genitourinary symptoms. There were no clinical features suggestive of connective tissue disease, however he had a significant family history of nasopharyngeal carcinoma. The patient has no prior history of TB and denies any known exposure to the disease. 

**Figure 1:**
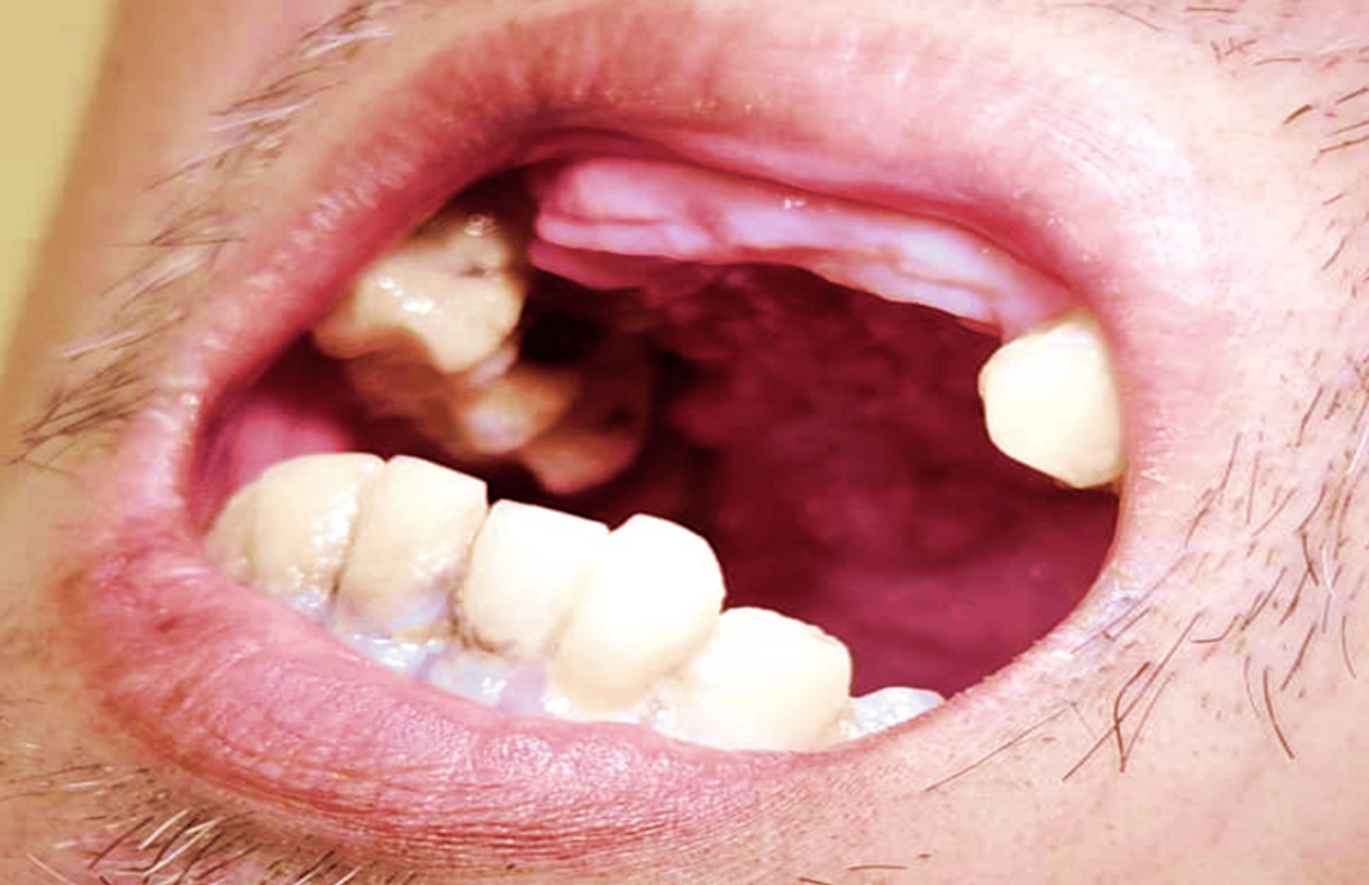
Ulcerated mass on the right hard palate, the mass has irregular edge and surface, 3cm x 4cm in size, erythematous, tender and no discharge.

**Figure 2:**
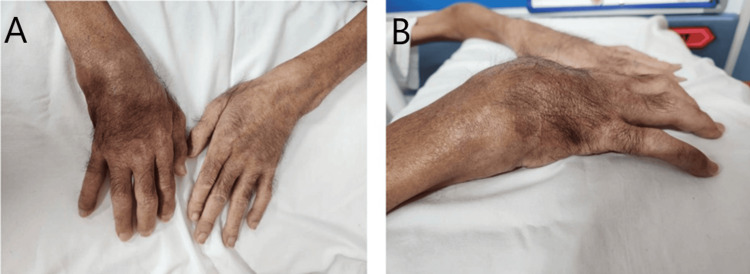
Right wrist swelling (A: anteroposterior view and B: lateral view), swollen, tender, reduced range of movement, with soft tissue swelling over dorsum.

On examination, he appeared cachectic with an ulcerated mass on the hard palate (Figure 1), without cervical lymphadenopathy. He had a left eye exotropia which was of uncertain duration. Scattered lung crepitations were noted, while cardiovascular and abdominal examinations were unremarkable. Synovitis of the right wrist with limited range of movement was observed (Figure 2), with no involvement of other joints. He was admitted the following day with a working diagnosis of palatal malignancy and suspected paraneoplastic monoarthritis. 

**Figure 3:**
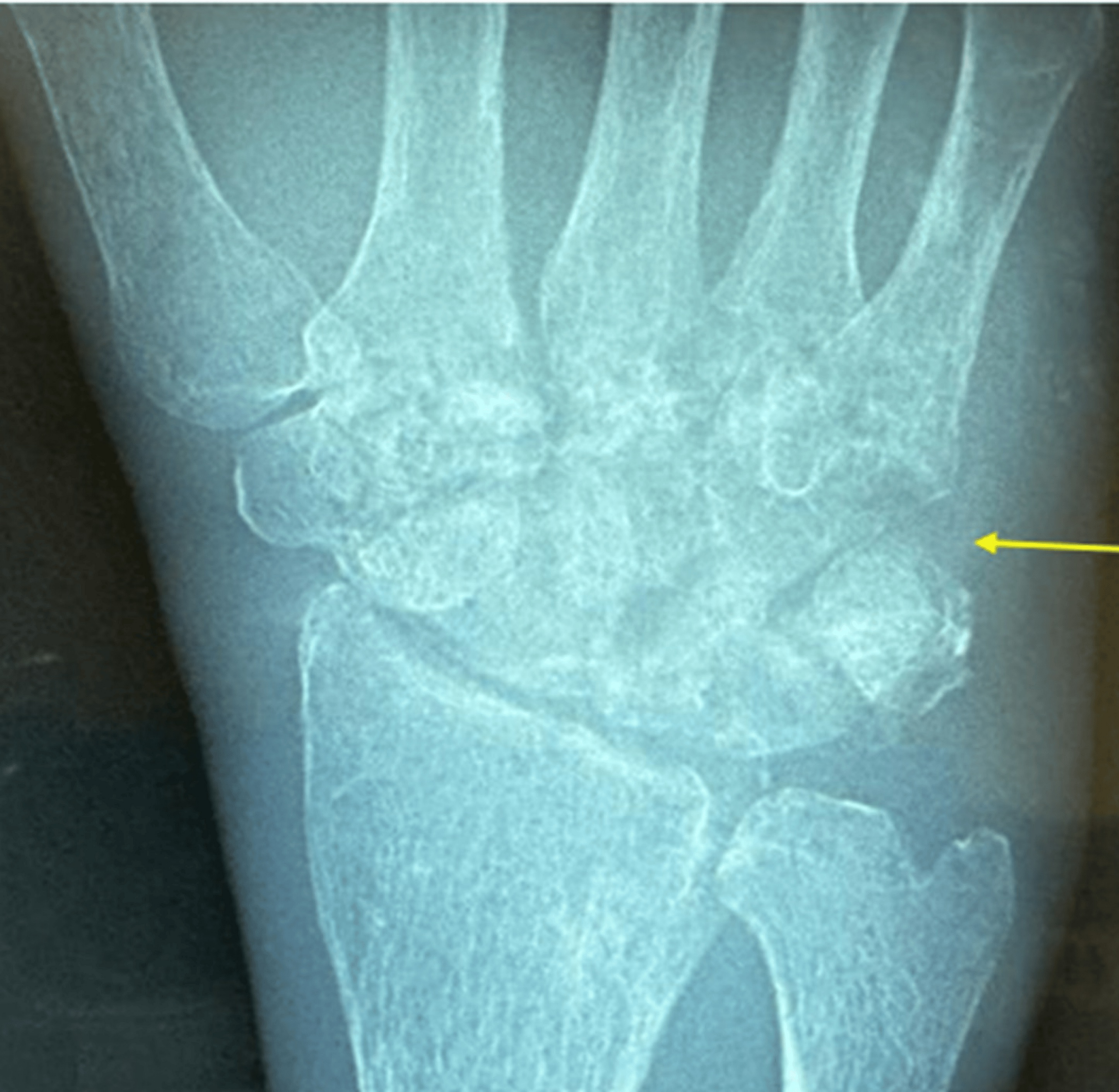
Hand X-ray (anteroposterior view) showed erosion of articular surfaces of the second to fifth metacarpal bone and distal articular surface of the adjacent carpal bones (yellow arrow), reduced radiocarpal joint space, with background of osteopenic bone, no fracture or dislocation.

**Figure 4:**
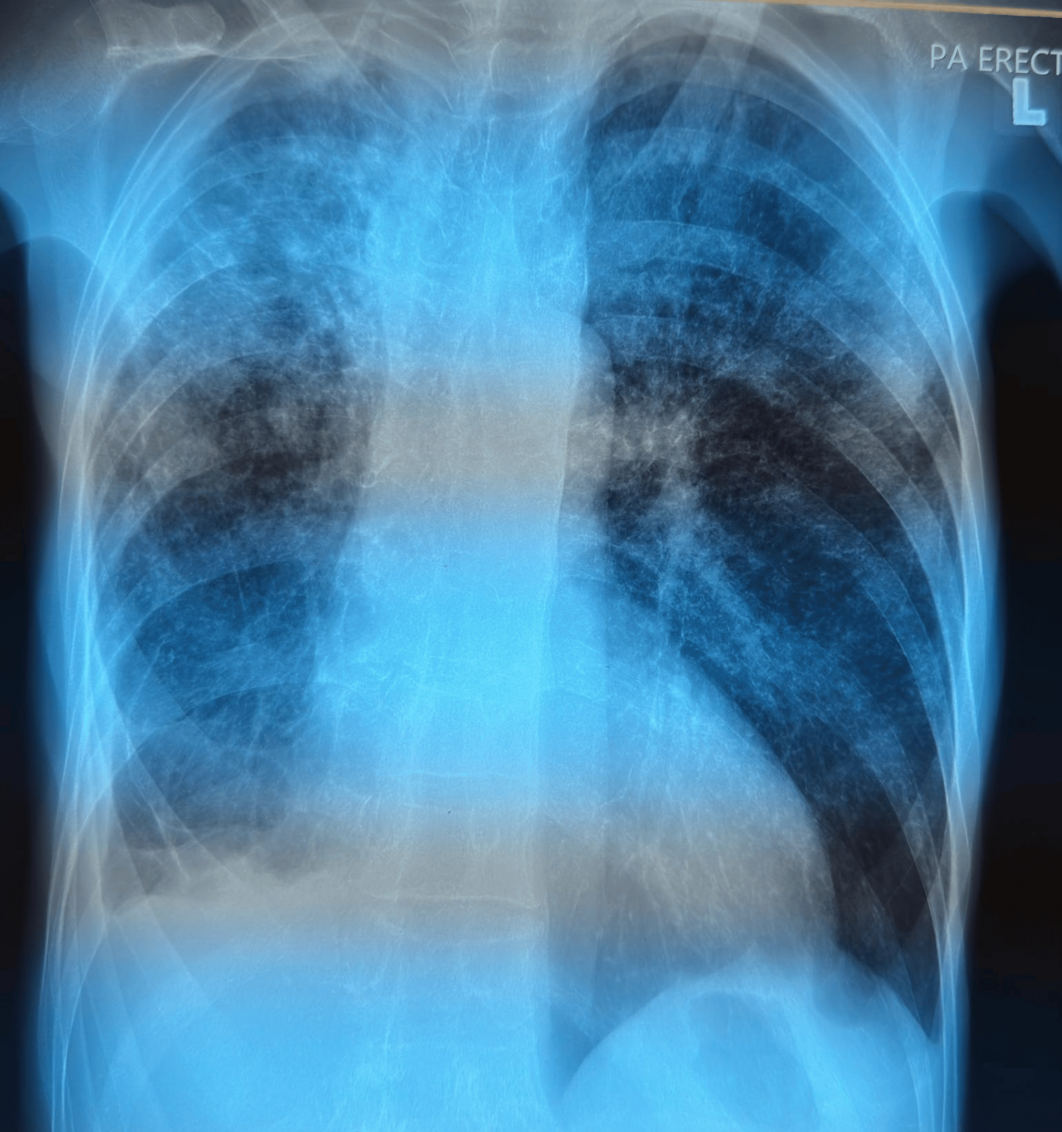
Chest X-ray (posteroanterior erect) showed reduced right lung volume, with fibrotic changes over lung bases and right apical region. Diffuse reticular nodular opacities in both lung fields.

Laboratory investigations revealed normocytic normochromic anemia, thrombocytosis (likely due to reactive cause), elevated C-reactive protein (CRP) and erythrocyte sedimentation eate (ESR), hypercalcemia, hypoalbuminemia, and renal impairment (Table 1). Radiograph of the right wrist demonstrated erosive changes of the carpal and carpometacarpal joints (Figure 3), while chest X-ray showed reticulonodular lung opacities in bilateral lung fields, and fibrotic changes in the right upper zone (Figure 4).

**Table 1:** Blood investigation taken during his admission. ALT: alanine aminotransferase; AST: aspartate aminotransferase

Parameters	Patient's Value	Reference Range
Hemoglobin (g/dL)	8	13-17
Total White Cell (10^9/L)	10	11
Platelet (10^9/L)	455	150-450
Urea (mmol/L)	18	3-8
Creatinine (umol/L)	224	50-90
Albumin (g/L)	25	32-48
Globulin (g/L)	32	25-39
Total Bilirubin (umol/L)	4.5	<24
AST (U/L)	35	10-50
ALT (U/L)	40	10-50
Corrected Calcium (mmol/L)	3.41	2-2.6
Erythrocyte Sedimentation Rate (mm/Hr)	120	<20
C-Reactive Peptide (mg/L)	102	<5
Fasting Blood Sugar (mmol/L)	4.2	3.9-5.5
HbA1c (%)	5.3	<6.3
HIV antibody	non reactive	
Hepatitis B surface antigen	non reactive	
Hepatitis C total antibody	non reactive	
Blood Culture and Sensitivity	no growth	

**Figure 5:**
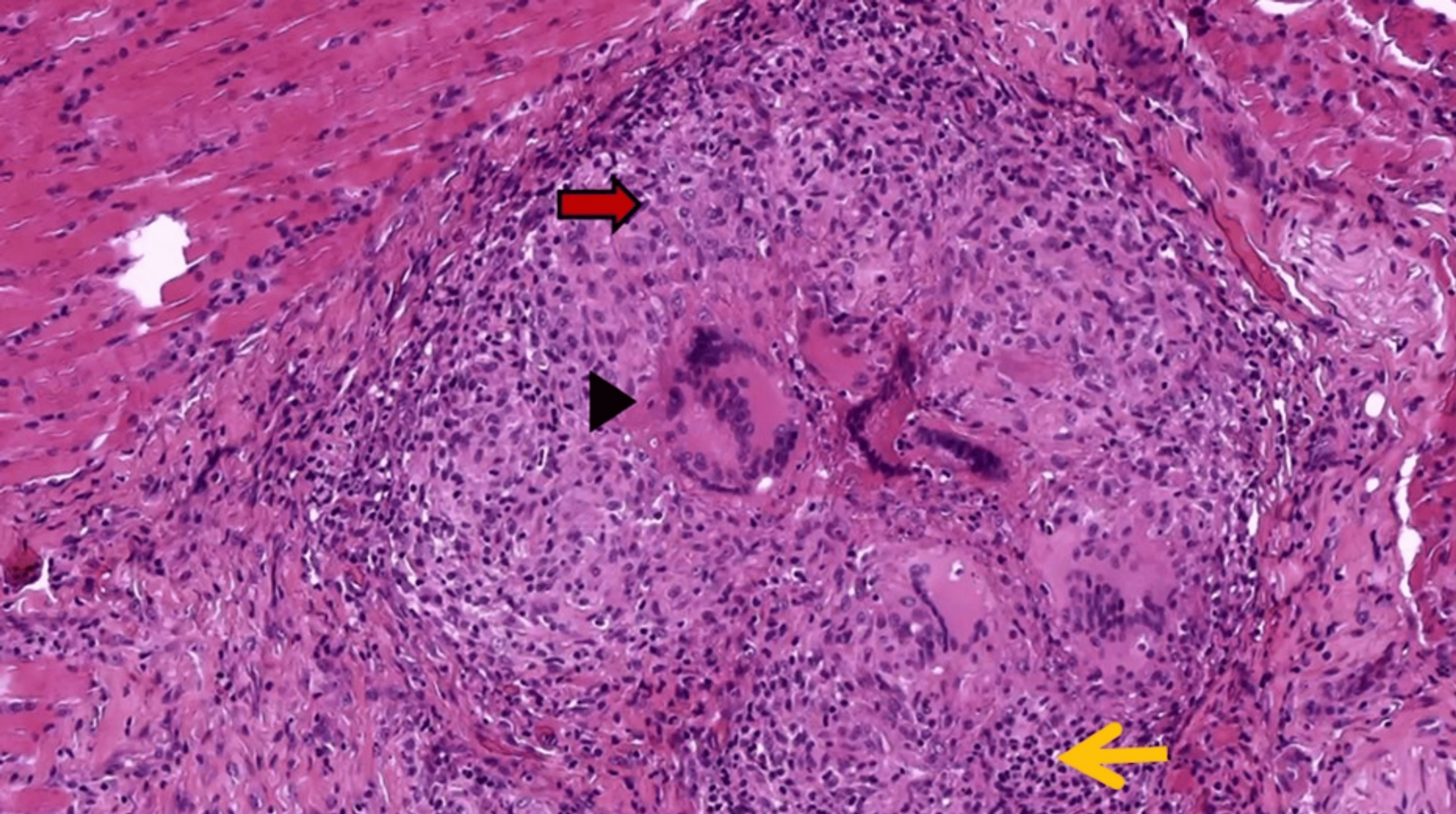
Palatal mass - Hematoxylin & Eosin (H&E) stain demonstrates granulomatous inflammation composed of histiocytes (red arrow), multinucleated giant cell (arrowhead) and lymphocytes (yellow arrow).

**Figure 6:**
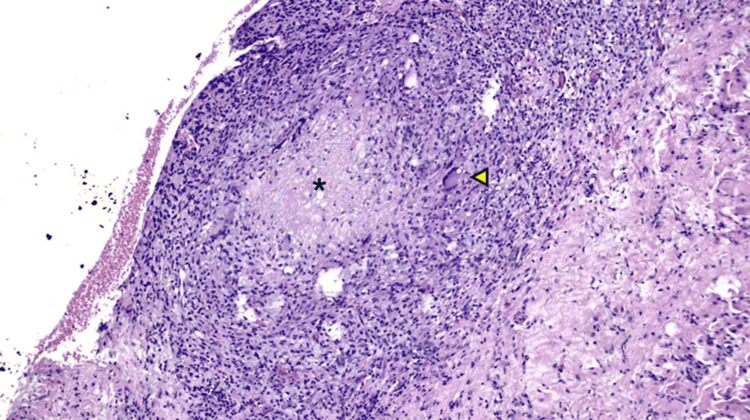
Granulomatous inflammation composed of epithelioid histiocytes, Langhans-type multinucleated giant cells (yellow arrowhead), and lymphocytes (x100). Central caseous necrosis (asterisk) was also noted.

**Figure 7:**
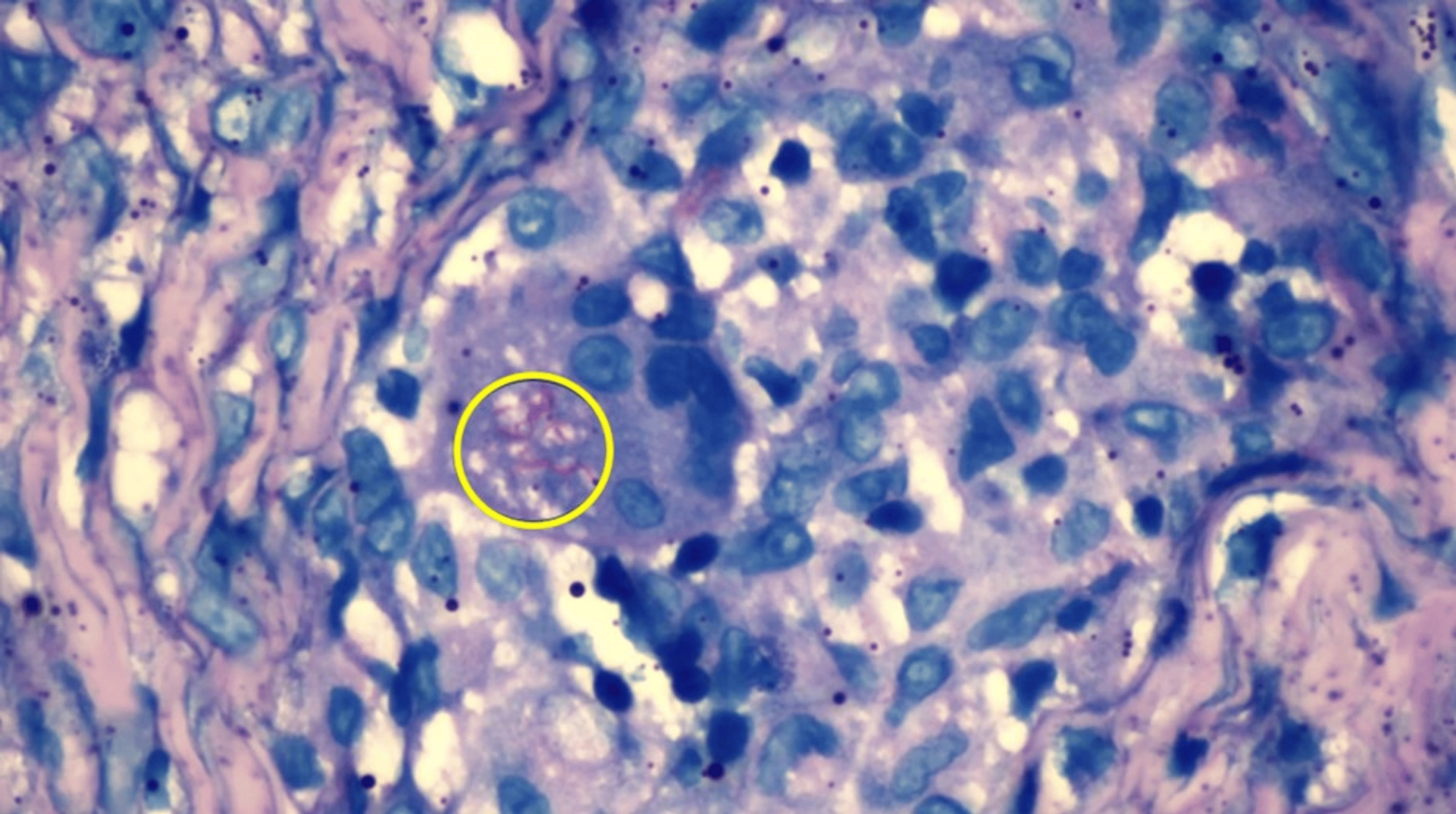
Ziehl-Neelsen stains of palatal mass highlight the presence of acid-fast bacilli (yellow circle).

Supportive treatment with intravenous fluids, analgesia, and oxygen supplementation was initiated. Given the suspicion of malignancy and greater accessibility of the palatal lesion, an incisional biopsy was performed, with the presumption that the wrist involvement was paraneoplastic. Wrist biopsy was deferred. A non-enhanced brain computerized tomography (NECT) was performed for left exotropia, which showed a left eye staphyloma without focal brain lesion; however, further evaluation, such as contrasted imaging could not be completed due to the patient's clinical instability. On day 4 of admission, the patient started to develop fever, prompting evaluation for TB. He was started on a broad-spectrum antibiotic (amoxicillin clavulanate). As he was unable to produce sputum sample for examination despite being induced, nasogastric lavage was obtained, which tested positive for acid-fast bacilli (AFB). Histopathology of the palatal biopsy subsequently demonstrated central caseous necrosis, with granulomatous inflammation, and presence of AFB in Ziehl-Neelsen stains consistent with TB (Figures 5-7), with no evidence of malignancy.

Anti-TB therapy consisting of rifampicin, ethambutol, isoniazid, and pyrazinamide was initiated in a timely manner on top of other standard of care management. Despite treatment, his condition deteriorated rapidly over a two-week hospital stay, complicated by pancytopenia and oliguric renal failure, requiring invasive mechanical ventilation, renal replacement therapy, and intensive care unit (ICU) admission. Further diagnostic procedures of right wrist were therefore limited by hemodynamic instability.

*Mycobacterium tuberculosis* isolated from nasogastric lavage and endotracheal aspirate cultures was pan-susceptible to first-line anti-TB agents; however, these results became available posthumously. This patient, who presented ill, continued to deteriorate despite treatment and eventually succumbed to multiorgan failure, highlighting the aggressive course of disseminated TB in this immunocompetent host. This case underscores the importance of considering TB in the differential diagnosis of multisystem disease, particularly in endemic settings, even when malignancy appears more likely. The chronology of events is illustrated in Table 2. 

**Table 2:** Chronology of events TB: tuberculous, NECT: non-enhanced computed tomography; AFB: acid-fast bacilli, HPE: histopathological examination

Time	Event / Symptom	Investigation / Treatment
-6 months	Right hand pain, intermittent fever, anorexia, weight loss	Self-purchase analgesia
-2 months	Difficulty in chewing and swallowing	
Day 0	Presented to Rheumatology Clinic	
Day 1	Admitted to ward to work up for palatal mass	Supportive treatment – hydration, oxygen therapy, analgesia
Day 2	Biopsy of palatal mass	
Day 4	Left exotropia, Fever	NECT brain: left eye staphyloma, no focal brain lesion. Broad-spectrum antibiotics started
Day 5		Nasogastric lavage taken
Day 6	Desaturated	Non-invasive ventilation. Nasogastric lavage: positive for AFB, HPE of palatal mass confirmed TB. Anti-TB initiated
Day 7	Further deterioration, required inotropic support	Invasive mechanical ventilation, admitted to ICU, started on IV hydrocortisone (sepsis)
Day 8 – 11	Oliguric kidney failure	Renal replacement therapy (acute hemodialysis)
Day 12	Pancytopenia and transaminitis	Blood Culture & Sensitivity throughout admission: No growth
Day 13	Succumbed to illness	
2 months		TB Culture & Sensitivity of nasogastric lavage and endotracheal aspirate: *Mycobacterium tuberculosis* pan-susceptible to first-line anti-tuberculous agents

## Discussion

This case presents an unfortunate but instructive example of disseminated TB manifesting through two unusual features: ulcerated oral mass and erosive wrist arthritis. While pulmonary TB is common infectious disease, this case reminds us how EPTB can mimic other conditions, often leading to delays in diagnosis and treatment.

This diagnostic pitfall is well documented; oral TB lesions frequently mimic malignancy or fungal infections. The marked disparity in incidence between oral squamous cell carcinoma and oral TB means that ulcerated oral lesions are frequently misinterpreted as malignancy, resulting in delayed diagnosis of this rare infectious entity. A useful distinguishing feature is that palatal TB typically presents as a painful ulcer or mass [2], whereas oral cancer often appears as a painless, non-healing ulcer or exophytic growth in its early stages, potentially progressing to cause pain, bleeding, or dysphagia. 

The oral manifestation in our patient proved particularly challenging. TB of the oral cavity is exceptionally rare, accounting for less than 1% of all extrapulmonary TB cases [3]. It usually results from secondary inoculation of mucosa compromised by ulceration, trauma, or contact with infected sputum from another site. The most commonly affected oral sites include the tongue, followed by the palate, lips, buccal mucosa, and gingiva [4]. In this case, the ulcerated lesion on the hard palate was especially concerning given the patient's family history of nasopharyngeal carcinoma, initially raising strong suspicion of malignancy. However, a biopsy ultimately revealed characteristic granulomata with presence of AFB consistent with TB.

The patient's wrist involvement added an intriguing dimension to the case. While spinal TB (Pott’s disease) is the most common form of skeletal TB, non-spinal osteoarticular TB, including TB arthritis, is considerably rarer and typically presents insidiously, making early diagnosis challenging. TB arthritis most commonly affects large, weight-bearing joints such as the knees and hips; however, disseminated disease can manifest in atypical locations as illustrated in this case. 

Wrist joint TB is a rare subtype of musculoskeletal TB, accounting for less than 1% of all osteoarticular TB cases [5]. The infection often originates in the tendon sheath and subsequently spreads to adjacent joint capsule, muscle, tendon and synovium, potentially leading to tissue necrosis, tendon adhesions, and functional impairment [6]. Diagnosis is especially difficult due to the non-specific nature of its clinical presentation. Pain and swelling are the usual initial symptoms.

In this patient, the absence of classic systemic features such as persistent cough and fever coupled with presence of a family history of nasopharyngeal carcinoma misled the clinical impression toward malignancy-until a chest X-ray suggested TB, later confirmed via nasogastric aspirate positive for AFB. Wrist aspiration and biopsy was deferred in favour of the more accessible palatal lesion, with presumed concordance in pathology; definitive evaluation was not achieved due to hemodynamic instability. Ideally, had clinical stability permitted, further studies including aspiration, advanced imaging, and biopsy would have been performed. 

In a case series conducted in the United States, synovial fluid analysis often reveals high leukocyte counts (10,000-50,000/μL) with lymphocyte predominance. AFB smears are positive in only about one-fifth of patients, reflecting the paucibacillary nature of TB arthritis. PCR testing for TB is highly specific in synovial fluid analysis. In contrast, cultures are positive in nearly 80% of confirmed cases [7]. Biopsies of bone lesions, synovium, or soft tissue provide critical evidence in establishing a diagnosis of TB. 

Imaging, while generally non-specific, is crucial for early detection and evaluation. Radiographs may show an ill-defined wrist mass, bone destruction, joint space narrowing, and involvement of the distal radius and ulna. The classic triad known as Phemister’s triad - juxta articular osteoporosis, peripheral erosions, and gradual joint space narrowing - is suggestive of TB arthritis [8]. CT imaging is especially valuable for assessing bone destruction and involvement of adjacent soft tissues. In wrist joint TB, CT scans can reveal bony deformities and patchy bone density changes [9]. Magnetic Resonance Imaging (MRI) is highly effective for detecting osteoarticular TB, particularly through visualization of synovial thickening, soft tissue involvement, and differentiation between infected and healthy tissue [10]. The combined use of MRI and CT offers a comprehensive evaluation of joint integrity and periarticular extension.

This patient’s metabolic complications may provide important diagnostic clues. His significant hypercalcemia and acute kidney injury could reflect systemic effects of granulomatous inflammation, a process in which activated macrophages can increase production of active vitamin D [11]. Although vitamin D levels were not measured, this mechanism is a plausible contributor. When such metabolic disturbances occur alongside oral lesions and arthritis, TB or sarcoidosis should be considered.

Despite eventually confirming the diagnosis through AFB-positive nasogastric lavage, the patient's rapid clinical deterioration and death highlight the devastating consequences of delayed TB diagnosis. The literature suggests mortality rates of approximately 25-30% in delayed diagnoses [12].

## Conclusions

This case demonstrates disseminated TB presenting with unusual extrapulmonary manifestations, including an ulcerated palatal mass and suspected wrist involvement, initially mimicking malignancy and inflammatory arthritis. The diagnosis was confirmed through histopathological examination of the palatal mass and positive mycobacterial cultures from respiratory specimens, highlighting the value of accessible diagnostic sites when direct sampling of all affected regions is not feasible.

Despite appropriate therapy, the patient experienced rapid clinical deterioration, underscoring the potential for fulminant disease even in immunocompetent individuals. While generalizable conclusions cannot be drawn, this case adds to the spectrum of reported atypical manifestations and reinforces the need for a high index of clinical suspicion in similar presentations. 
